# Canonical Wnt signaling protects hippocampal neurons from Aβ oligomers: role of non-canonical Wnt-5a/Ca^2+^ in mitochondrial dynamics

**DOI:** 10.3389/fncel.2013.00097

**Published:** 2013-06-25

**Authors:** Carmen Silva-Alvarez, Macarena S. Arrázola, Juan A. Godoy, Daniela Ordenes, Nibaldo C. Inestrosa

**Affiliations:** ^1^Departamento de Biología Celular y Molecular, Centro de Envejecimiento y Regeneración, Facultad de Ciencias Biológicas, Pontificia Universidad Católica de ChileSantiago, Chile; ^2^Laboratorio de Neurobiología Molecular, Departamento de Biología Celular y Molecular, Facultad de Ciencias Biológicas, Pontificia Universidad Católica de ChileSantiago, Chile

**Keywords:** Wnt-5a signaling, Wnt/ Ca^2+^, mitochondria, Aβ oligomers, hippocampal neurons

## Abstract

Alzheimer's disease (AD) is the most common type of age-related dementia. The disease is characterized by a progressive loss of cognitive abilities, severe neurodegeneration, synaptic loss and mitochondrial dysfunction. The Wnt signaling pathway participates in the development of the central nervous system and growing evidence indicates that Wnts also regulate the function of the adult nervous system. We report here, that indirect activation of canonical Wnt/β-catenin signaling using Bromoindirubin-30-Oxime (6-BIO), an inhibitor of glycogen synthase kinase-3β, protects hippocampal neurons from amyloid-β (Aβ) oligomers with the concomitant blockade of neuronal apoptosis. More importantly, activation with *Wnt-5a*, a non-canonical Wnt ligand, results in the modulation of mitochondrial dynamics, preventing the changes induced by Aβ oligomers (Aβo) in mitochondrial fission-fusion dynamics and modulates Bcl-2 increases induced by oligomers. The canonical Wnt-3a ligand neither the secreted Frizzled-Related Protein (sFRP), a Wnt scavenger, did not prevent these effects. In contrast, some of the Aβ oligomer effects were blocked by Ryanodine. We conclude that canonical Wnt/β-catenin signaling controls neuronal survival, and that non-canonical Wnt/Ca^2+^signaling modulates mitochondrial dysfunction. Since mitochondrial dysfunction is present in neurodegenerative diseases, the therapeutic possibilities of the activation of Wnt signaling are evident.

## Introduction

Wnt proteins are involved in regulating axon guidance, dendrite morphogenesis, and synapse formation (Inestrosa and Arenas, 2010; Budnik and Salinas, 2011; Park and Shen, 2012). Wnts have also been implicated in synaptic plasticity and modulation of long-term potentiation (LTP) in mouse hippocampal slices (Chen et al., 2006; Cerpa et al., 2011). Moreover, the expression of Wnts in the mature nervous system suggests that Wnt signaling plays a key role in neuroprotection and synaptic plasticity (De Ferrari and Moon, 2006; Toledo et al., 2008).

The canonical Wnt pathway is activated by the binding of the ligand to its receptor, Frizzled (Fz), leading to glycogen synthase kinase-3β (GSK-3β) inactivation and the dissociation of β-catenin from the destruction complex (Gordon and Nusse, 2006; Angers and Moon, 2009). Under these conditions, β-catenin is accumulated in the cytoplasm and translocate to the nucleus where it associates with the TCF/LEF transcription factor and regulates Wnt target gene expression (Arrázola et al., 2009; Inestrosa et al., 2012; Nusse and Varmus, 2012). This pathway plays a key role in pre-synaptic assembly of central synapses (Ahmad-Annuar et al., 2006; Cerpa et al., 2008). There are at least two β-catenin-independent pathways: the planar cell polarity (PCP) pathway and the Ca^2+^ pathway. The PCP pathway regulates tissue polarity and cell migration, and it is known as the Wnt/JNK pathway. The activation of the Wnt/Ca^2+^ pathway triggers the increase in intracellular Ca^2+^ levels and activates the protein kinases Ca^2+^/Calmodulin-dependent protein kinase II (CamKII) and protein kinase C (PKC) (Toledo et al., 2008; Angers and Moon, 2009).

At the post-synaptic compartment, Wnt signaling modulates the assembly of the post-synaptic apparatus (Farías et al., 2009; Varela-Nallar et al., 2010). It was observed that activation with *Wnt-5a* induces rapid changes in the clustering of the post-synaptic density protein (PSD-95), through a JNK-dependent signaling pathway, indicating that the Wnt-5a/JNK pathway modulates the post-synaptic region of the mammalian synapse (Farías et al., 2009).

Neurons are highly dependent on mitochondrial function for energy supply. Mitochondria in neurons are dynamic; they can migrate, divide, and fuse. These processes are thought to facilitate energy distribution throughout neuronal projections and to sites of high-energy demand such as synapses, to maintain bioenergetic functionality (Westermann, 2010). In addition to energy supply, mitochondria also play a critical role in synaptic plasticity through the maintenance of calcium homeostasis in the synaptic microenvironment by calcium buffering. Mitochondria are remarkably dynamic and mitochondrial morphology is controlled by a dynamic balance between fission and fusion (Chan, 2012). The first observation of mitochondrial fission and fusion events was made in yeast (Nunnari et al., 1997; Hoppins et al., 2007). Regulation of mitochondrial division is critical for normal cellular function (Chan, 2012) and excess division is linked to numerous diseases, including neurodegenerative diseases like Alzheimer's disease (AD), Parkinson and Huntington (Cho et al., 2010; Johri and Beal, 2012; Manji et al., 2012; Itoh et al., 2013).

It has been previously demonstrated that Ca^2+^ influx leads to mitochondrial fission through activation of PKC, CaMKIα, and calcineurin, which activate Dynamin-related protein 1 (Drp1), a critical protein in mitochondrial dynamics (Smirnova et al., 2001; Qi et al., 2011). In fact, over the last few years, compelling evidence has demonstrated the relevance of the Wnt/Ca^2+^ pathway in several cellular processes (Kohn and Moon, 2005; Varela-Nallar et al., 2010). Considering the intracellular calcium increase, in response to the stimulation of the non-canonical Wnt/Ca^2+^ pathway, we previously analyzed and found that Wnt-5a might modulate mitochondrial dynamics through the possible activation of the Wnt/Ca^2+^ signaling pathway (Silva-Alvarez et al., submitted).

Here we investigated whether Wnt signaling protects neurons exposed to Aβ oligomers (Aβ o) as was previously demonstrated in the case of Aβ fibrils. Also, we studied the role of the non-canonical Wnt-5a ligand on mitochondrial fission-fusion, and in particular its effect on neurons exposed to Aβ oligomers. Wnt signaling protects neurons from Aβ oligomers, in particular, we found that Wnt-5a prevents changes in mitochondrial fission-fusion dynamics and also Bcl-2 exposure on the mitochondrial surface in rat hippocampal neurons.

## Materials and methods

### Primary cultured rat hippocampal neurons

Rat hippocampal cultures were prepared from Sprague-Dawley rats at embryonic day 18. At day 2, cultured neurons were treated with 2 μM cytosine arabinoside (AraC) for 24 h, to remove the number of glial cells. This method resulted in highly enriched neuron cultures (95% neurons) (Cerpa et al., 2008; Farías et al., 2009).

### Generation of control media and Wnt ligand conditioned media

Control and *Wnt* ligand conditioned media were prepared from L Cells (ATCC CRL-2648), L *Wnt-3* (ATTC CRL-2814) cells and L *Wnt-5a* (ATTC CRL-2814) cells. Cells were grown until 90% confluence, approximately, and the culture medium was replaced with neurobasal medium without supplement and antibiotics. After 60 h of incubation, the media was recovered, centrifuged, sterile filtered and stored at 4°C until use (Alvarez et al., 2004; Arrázola et al., 2009; Cuitiño et al., 2010).

### Formation of amyloid-β oligomers

Synthetic Aβ_1–42_ peptide corresponding to wild type human Aβ was obtained from Genemed Synthesis, Inc. (San Francisco, CA). Aβ peptide stock solution was prepared by dissolving freeze-dried aliquots of Aβ in 1,1,1,3,3,3-hexafluoro-2-propanol (HFIP, Sigma H-8508) at 1 mM, incubated at room temperature for 1 h, and lyophilized. For Aβo preparation, peptide film was dissolved in dimethyl sulfoxide (DMSO, Sigma D2650) at 5 mM and then diluted into distilled water to a final concentration of 100 μM. The preparation was incubated overnight for Aβ o formation (Klein, 2002). Aβ o were visualized by electron microscopy and analyzed by Tris-Tricine SDS gel electrophoresis, as previously described (Dinamarca et al., 2010, 2011).

### Neuronal viability assays

Hippocampal neurons plated on polylysine-coated coverslips (30,000 neurons/cover) were treated with different concentrations of Aβ o (1–20 μM) for 24 h. Live and dead neurons were analyzed in non-fixed cells with the LIVE/DEAD Viability/Cytotoxicity Kit (stock N° L3224) for mammalian cells (Molecular Probes, Carlsbad, CA).

### Mitochondrial length measurements

Hippocampal neurons were labeled with 50 nM Mitotracker Orange CMTMRos (Molecular Probes, M-7510) for 20 min at 37°C and photographed under confocal microscopy. Mitochondrial length was measured with Image J software (NIH). For comparison purposes mitochondria were classified into three different categories of length ranging from less than 0.5, 1–2, and greater than 3 μm. A minimum of 10 micrographs were made for each treatment and scored (Silva-Alvarez et al., submitted; Zolezzi et al., 2013).

### Immunofluorescence

Hippocampal neurons were plated on polylysine-coated coverslips (30,000 neurons/cover). Cells were rinsed twice in ice-cold PBS and fixed with a freshly prepared solution of 4% paraformaldehyde in PBS for 20 min, and permeabilized for 5 min with 0.2% Triton X-100 in PBS. After several rinses in ice-cold PBS, cells were incubated in 1% BSA in PBS (blocking solution) for 30 min at room temperature, followed by an overnight incubation at 4°C with primary antibodies. Cells were extensively washed with PBS and then incubated with Alexa-conjugated secondary antibodies (Molecular Probes) for 30 min at 37°C (12). *Primary antibodies:* rabbit anti-β-catenin (Santa Cruz Biotechnology, Inc.); mouse anti-β III-Tubulin (Promega Corporation); anti- Aβ 17–24 (4G8 clone)(EMD Millipore Corporation); and rabbit anti-Bcl2 (Cell Signaling Technology).

### Ryanodine (Ry) and thapsigargin incubation

Hippocampal neurons were incubated with blocking concentrations of Ry (20 μM) to inhibit Ry receptors (RyRs), since Ry could inhibit or activate RyRs in a concentration dependent manner (Adasme et al., 2011), or 5 μM of thapsigargin in order to inhibit SERCA, for different times, up to 5 h (Hom et al., 2007).

### Quantification and statistical analysis

The data represents the mean and SD or SEM from 4–6 independent experiments, where *n* stands for each independent experiment. *P*-values were obtained using Student's *t*-test. A *p*-value ^*^ < 0.05 was considered and was indicated on the graph by an asterisk. Error bars indicate SEM. ^*^p < 0.01; ^**^p < 0.001.

## Results

### Characterization of Aβo and their effect on neuronal viability

**Figure 1:**
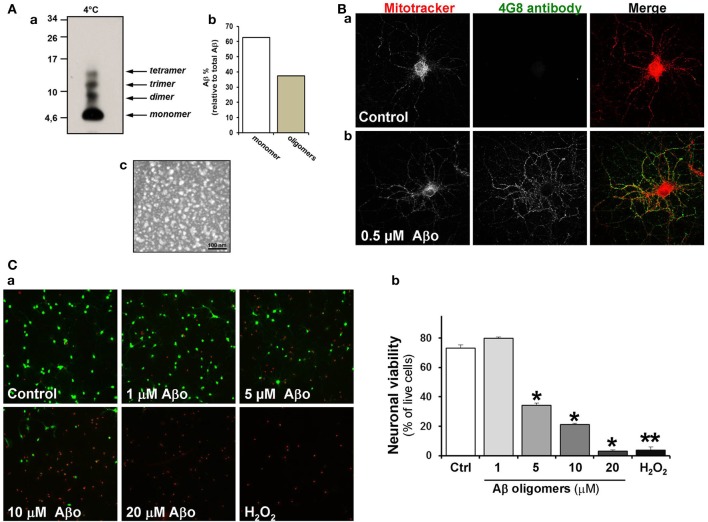
**Aβ oligomers are attached to the somatodendritic region of hippocampal neurons. (A)** Characterization of Aβ oligomer species. **a**, Different forms of Aβ o were analyzed by Tris-Tricine SDS gels using anti-4G8 antibody. **b**, Densitometric measurement represent relative percentage of oligomers to total Aβ. **c**, Electron microscopy shows the Aβ oligomer preparation obtained under negative staining, scale bar: 100 nm. **(B)** Hippocampal neurons were treated with Aβ oligomers and then stained with Mitotracker (red) and 4G8 antibody against Aβ_17–24_ (green). **a**, Control neurons; **b**, Neurons exposed to 500nM of Aβ oligomers for 24 h. (**C)** The viability of hippocampal neurons was measured in non-fixed cells using LIVE/DEAD kit assay. Neurons were treated for 24 h with various concentrations of Aβ oligomers (1–20 μM) **a**, Treated neurons were stained with Calcein-AM/EthD1. Calcein detects live cells (green) and ethidium stain dead cells (red). 0.5 mM H_2_O_2_ was used as a positive control for cell death **b**, Statistical analysis represents the neuronal viability using LIVE/DEAD assay. Results are the mean ± SEM. *n* = 3 experiments, Student's *t*-test, ^*^p < 0.05, ^**^p < 0.005.

Since some variability has been observed in various studies carried out so far with Aβ aggregates, we first characterized our Aβ o preparation. Figure 1A, shows a blot made using an anti-4G8 antibody where the pattern of dimers, trimers and tetramers were observed **(a)**. Roughly 50% of the initial monomer became oligomer **(b)**. Under examination using an electron microscope they appear as spheres, exactly as described previously (Dinamarca et al., 2010, 2011) **(c)**. In cultured hippocampal neurons, control and challenged with Aβ o, mitochondria were stained with Mitotracker and Aβ o were detected with the same antibody (4G8) as used in Figure 1B. The Aβ oligomers became attached to the somatodendritic region of pyramidal neurons, as previously described by Lacor et al. (2004). Our Aβ o preparation was proved to be neurotoxic with the Calcein/Ethidium method (Figure 1C, green, calcein stain; red, EthD1). Calcein and ethidium stains were decreased and increased, respectively, in neurons treated with 5 μM Aβ oligomers **(a)**. The quantification of viability decreased by 50% (35 ± 2%) when we used 5 μM Aβ o compared with the control (72 ± 3%) and when we used 20 μM Aβ o the viability decreased 90% (7 ± 1%) compared to the control (Figure 1Cb).

### A GSK-3β inhibitor, Bromoindirubin-30-Oxime (6-BIO), stabilizes β-catenin, and prevents neurotoxic effects of Aβ oligomers

**Figure 2:**
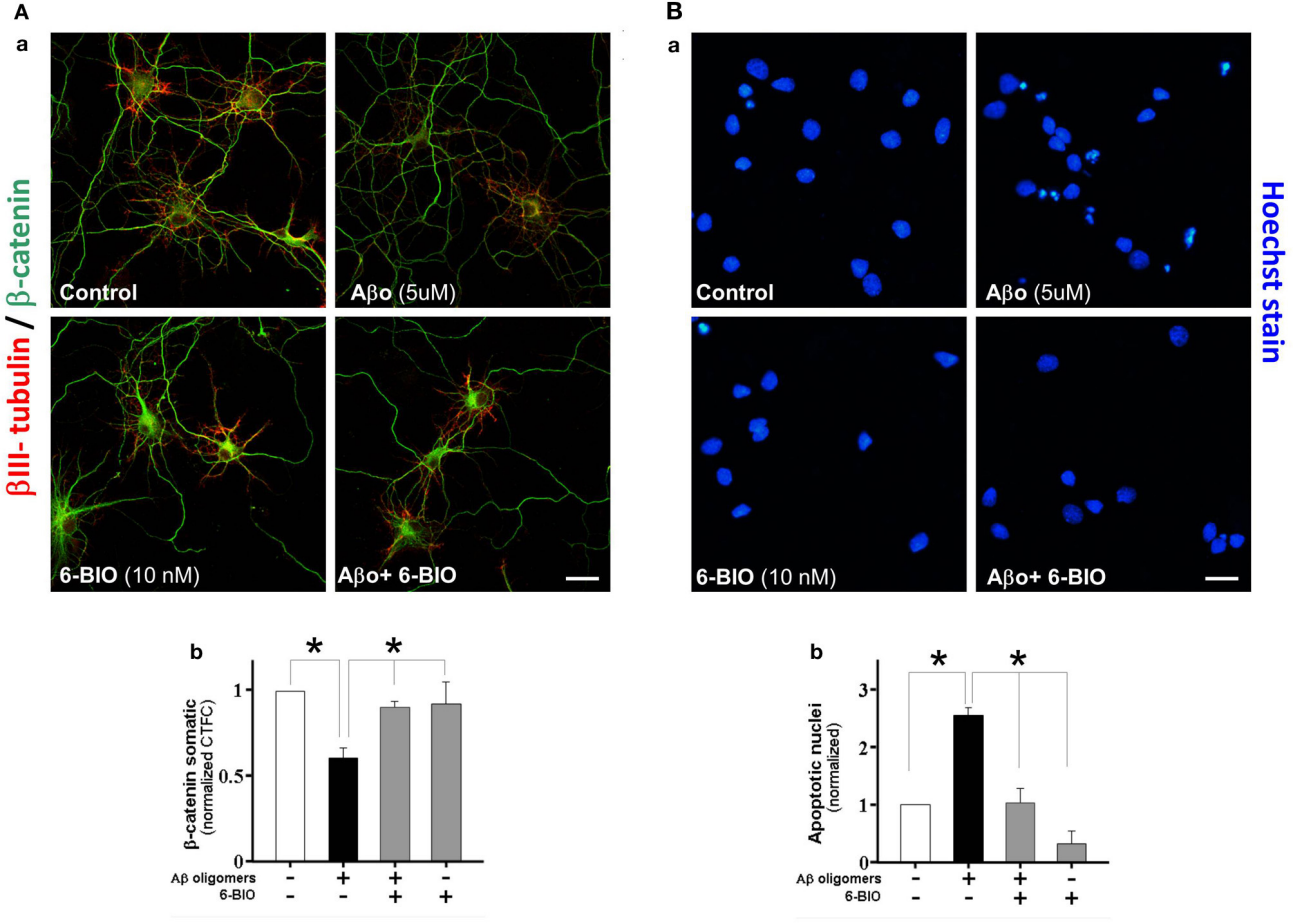
**Activation of canonical Wnt/β-catenin by a GSK-3b inhibitor (6-BIO) induces β-catenin stabilization and protects hippocampal neurons from Aβ oligomers damage. (A)** The micrographs show representative neurons stained with β III-tubulin (green) and β-catenin (red), **a**, control hippocampal neurons, and treatment with Aβ oligomers, 6-BIO or 6-BIO plus Aβ oligomers; **b**, the graph shows the somatic fluorescence of β-catenin in the neuronal soma. (**B)** The micrograph shows neurons stained with Hoechst to visualize apoptotic nuclei; **a**, Hippocampal neurons were treated under the same early conditions; **b**, the graph shows the number of apoptotic nuclei under conditions specified. Results are the mean ± SEM. *n* = 4–6 experiments, Student's *t*-test, ^*^p < 0.05. Bar represent 10 μm.

Earlier work in our laboratory (Alvarez et al., 2004; Farías et al., 2007) showed that activation of the canonical Wnt signaling pathway with either Wnt-3a or Wnt-7a in hippocampal neurons prevents the neurotoxicity triggered by Aβ aggregates formed by amyloid fibrils. In the present study, we assessed the capacity of canonical Wnt signaling activation to prevent the neurodegenerative effects of Aβ oligomers. In these experiments, to activate the canonical Wnt signaling pathway, we used 6-BIO an inhibitor of GSK-3β at low concentrations (10 nM) (Meijer et al., 2004; Polychronopoulos et al., 2004). In cultured neurons we evaluated the integrity of neuronal branching with β III-tubulin and β-catenin levels under treatment of 5 μM Aβ oligomers plus 10 nM 6-BIO. This compound prevented almost all the Aβ oligomer-induced neurotoxicity and also recovered β-catenin levels in the neuritic branches and particularly at the soma, as shown in the graph (Figure 2A, graph: b). In addition, we also studied the effect of Aβ oligomers and 6-BIO on apoptosis, using Hoechst staining. In hippocampal neurons treated with 5 μM of Aβ oligomers, several picnotic nuclei were observed (Figure 2Ba), however, the apoptotic nuclei were not observed when 6-BIO was present. The quantification shows a 2.5 fold increase in apoptotic nuclei over the control, and this phenomenon was decreased to control levels when 6-BIO was present (Figure 2Bb). These studies with 6-BIO indicate that GSK-3β inhibition induces β-catenin stabilization and the consequent activation of the canonical Wnt/β-catenin signaling, which in turn protects neurons from the apoptotic effects of Aβ oligomers.

### Wnt-5a modulates mitochondrial dynamics on hippocampal neurons

**Figure 3:**
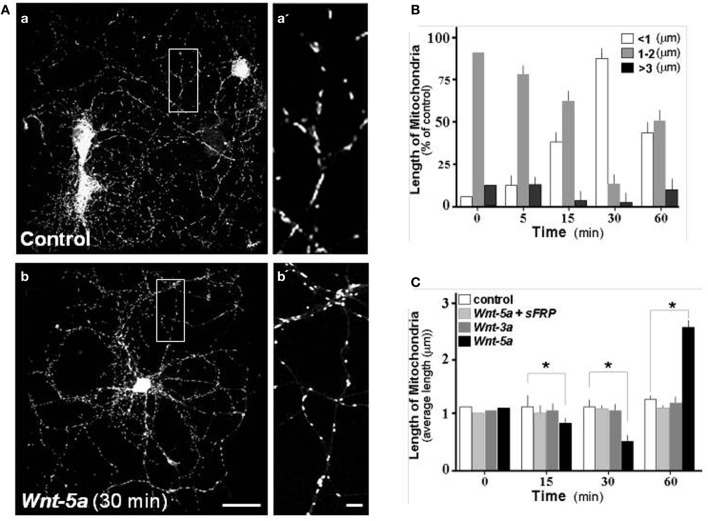
**Wnt-5a modulates mitochondrial dynamics in hippocampal neurons.** Hippocampal neuron cultures of 15 days *in vitro* were labeled with Mitotracker and treated with different Wnt ligands. **(A)** Photographs show mitochondria staining with Mitotracker in control and treated neurons, **a**, Control neurons; **a'**, magnification of mitochondria, **b**, treated with Wnt-5a, **b'**, magnification of mitochondria treated with Wnt-5a for 30 min. **(B)** Various populations of mitochondria were selected and measured in control and treated neurons. The graph shows the average mitochondrial length distribution observed in a time course experiment with Wnt-5a. The quantitative analysis considered a mitochondrial length ranging from <1 μm (white bars), 1–2 μm (gray bars) and greater than 3 μm (black bars). **(C)** Quantitative analysis of mitochondrial dynamics in neurons treated with different ligands. The graph shows the average mitochondrial length distribution observed in a time course treatment with control (white bars), *Wnt-5a* plus sFRP (light gray bars), Wnt-3a (dark gray bars), Wnt-5a (black bars), bar **(a,b)**, 10 μm; bar **(a',b')**, 1 μm. Results are the mean ± SEM. *n* = 4–6 experiments, Student's *t*-test, ^*^p < 0.05.

Changes in mitochondrial morphology was quantified by measuring the length of mitochondria stained with Mitotracker Orange, a reduced probe that fluoresces only when it enters living cells, where it is oxidized and then sequestered into the mitochondria. This is why it is widely used as a mitochondrial membrane potential (MMP) indicator. Pictures were obtained using a confocal fluorescence microscope. As discussed in the Materials and Methods section, we selected three types of Mitotracker-stained particles according to their different lengths: less than 0.5 μm representing mitochondria undergoing fission; particles of 1–2 μm that represent mitochondrial intermediate length in equilibrium between fission-fusion processes; and particles of more than 3 μm representing long mitochondria after the fusion process (Zolezzi et al., 2013). In control hippocampal neurons 15 days *in vitro* (DIV), after 2 h in control medium, mitochondria stained with Mitotracker showed an intermediate size morphology (Figure 3A, magnification in **a′**), however when neurons were treated with Wnt-5a for 30 min (Figure 3Ab), mitochondria looked smaller which is consistent with morphological changes related to fission events (Figures 3Aa, vs. 3Ab; Figure 3B, white bar 90 ± 8%). Later, after 1 h of incubation with Wnt-5a, a fusion process became apparent (Silva-Alvarez et al., submitted). When neurons were co-incubated with Wnt-5a plus a scavenger sFRP (Rattner et al., 1997), the mitochondrial changes observed in the presence of Wnt-5a alone were abolished (Figure 3C: light gray bar). At the same time, neurons exposed to a canonical control Wnt ligand, Wnt-3a, showed no change in mitochondrial morphology (Figure 3C: gray bar). These results suggest that mitochondrial dynamics are specifically stimulated by the Wnt-5a ligand (Figure 3C: dark bar; 15 min, 0.75 ± 0.15; 30 min, 0.4 ± 0.15; 60 min, 2.5 ± 0.2 μm).

### Wnt-5a protects mitochondria from damage by Aβ oligomers

**Figure 4:**
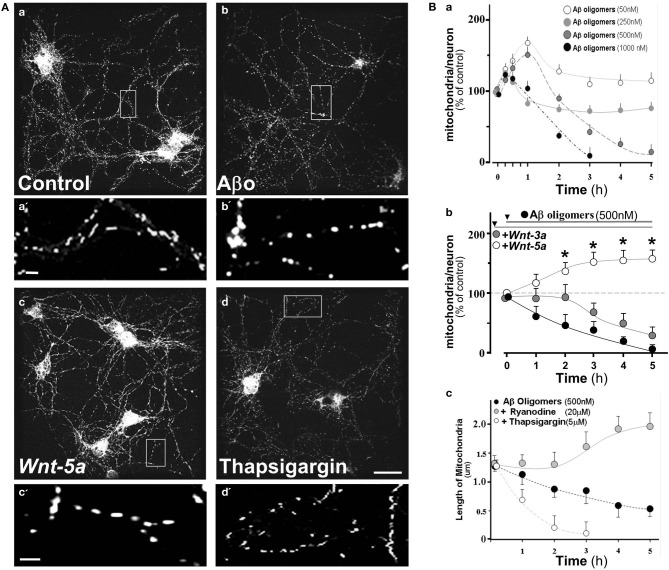
**Aβ oligomers modify mitochondrial dynamics in hippocampal neurons, Wnt-5a ligand protects and stabilizes mitochondrial membrane potential from such effects.** 15 day *in vi*tro hippocampal neuron cultures were labeled with Mitotracker orange and treated with various concentrations of Aβ oligomers. (**A)** Photographs show mitochondria stained with Mitotracker in control and treated neurons, **a**, Control neurons; **a**‘, magnification of mitochondria; **b**, treated with Aβ oligomers; **b'**, magnification of mitochondria treated with Aβ oligomers, **c**, treated with Wnt-5a, **c'**, magnification of mitochondria treated with Wnt-5; **d**, treated with 5 μM Thapsigargin. **(B)** Quantitation of mitochondrial dynamics under different conditions; **a**, Graph shows mitochondria/neuron ratio in a time course experiment under various concentrations of Aβ oligomers; **b**, Neurons pretreated with Wnt-5a or Wnt-3a and then challenged with Aβ oligomers; **c**, Length of mitochondria under inhibitors of RyR and SERCA. Primary cultures of rat embryo hippocampal neurons (15 DIV) were treated at 37°C. bar **(a,b,c,d)**, 10 μm; bar **(a',b',c')**, 2 μm. Results are the mean ± SEM. *n* = 4–6 experiments, Student's *t*-test, ^*^p < 0.05.

Considering Aβ-mediated neurotoxicity in AD (Haass and Selkoe, 2007; Ballard et al., 2011), we evaluated the effect of Aβ o and the participation of Sarco/Endoplasmic reticulum Ca^+2^-ATPases (SERCA) in this process. We exposed hippocampal neurons to 0.5 μM Aβ oligomers for 2 h. Similarly, cultures were exposed to *Wnt-5a* and 5 μM Thapsigargin, a SERCA inhibitor, for the same period of time. Mitochondria were stained with Mitotracker and examined using a Confocal Microscope. As expected, Aβ oligomers induced a significant alteration of the size of the mitochondrial population compared to the control (Figure 4Aa vs. 4Ab), with an increase in the number of small rounded mitochondria (see magnification **a′**, **b′**). After treatment with Wnt-5a, mitochondria looked rather normal (Figure 4Ac), however in the presence of Thapsigargin, only small sized mitochondria were observed, indicating some degenerative changes (Figure 4Ad) (see also magnification **c′** and **d′**), as this has been described previously in ER-calcium release studies (Jiang et al., 1994; Hom et al., 2007; Friedman et al., 2010; San Martín et al., 2012).

To examine whether or not the effects of Aβ o on the number of mitochondria were concentration dependent, hippocampal neurons were incubated with increasing amounts of Aβ o (Figure 4Ba). After 5 h of treatment with 50 nM Aβ o, almost no effect was observed, however, a transient increase was apparent at 1 h. In addition, a rather small change was observed with 250 nM Aβ oligomers. However, at 0.5 and 1.0 μM of Aβ o, a clear decrease of the mitochondria/neuron ratio was apparent (Figure 4Ba, 3 h: 40 ± 5, 6.5 ± 5%), probably due to the reduction of MMP in response to Aβ o, and therefore a decrease in the number of functional mitochondria. In order to compare the putative protective effect of Wnt ligands, neurons were pre-incubate with Wnt-3 and Wnt-5a ligands by separate, before 0.5 μM Aβ o challenge (Figure 4Bb). Wnt-5a, a non-canonical Wnt ligand, clearly protected from the toxic effect of the Aβ oligomers from very early on and the mitochondria/neuron ratio increased almost 50% after 3 h of co-treatment (Figure 4Bb, white circles, 3 h: 152 ± 5%). This suggests that Wnt-5a protects or increases the number of functional mitochondria in neurons. This effect was sustained over time and was also capable of preserving MMP in these neurons during the analyzed period. A different situation was observed with Wnt-3a, a canonical *Wnt* ligand, which during the first 2 h of treatment was able to prevent the Aβ o effect, however, after 3 h a similar decay to the one observed with oligomers treatment was apparent (Figure 4Bb, gray circles, 3 h: 75 ± 5%), indicating that canonical Wnt signaling was not able to sustain a long-term protection/preserving MMP of these mitochondria. Previous evidence indicates that the Wnt-3a ligand enhances mitochondrial biogenesis through Insulin receptor substrate-1 (IRS-1) producing, as a consequence, an increase of reactive oxygen species (ROS) levels and oxidative damage in non-neuronal cells (Yoon et al., 2010).

Early studies carried out in our laboratory indicate that Wnt-5a induces a rapid increase in intracellular calcium, as predicted by the Wnt/Ca^2+^ pathway (Varela-Nallar et al., 2010). The main targets of intracellular calcium are the SERCA and the Ryanodine receptor (RyR), both located in the ER, that control the mitochondrial calcium and the local feedback of intracellular calcium homeostasis (Paula-Lima et al., 2011). To evaluate the role of these receptors, we used specific inhibitors for each one, including Thapsigargin (SERCA) and Ryanodine (RyR).

The graph shows the mitochondria length in the presence of RyR and SERCA inhibitors in a time-course experiment (Figure 4Bc). Aβ oligomers decreased mitochondrial length, held constantly during the 5 h period of study (Figure 4Bc: black circle, 0.55 ± 0.2 μm), however when we blocked RyR (20 μM Ryanodine), the mitochondrial length appeared normal and eventually increased (Figure 4Bc: gray circle, 5 h: 1.9 ± 0.2 μm), suggesting that Ryanodine blocks the mitochondrial fragmentation induced by Aβ o (Paula-Lima et al., 2011). When the hippocampal neurons were co-incubated with Aβ oligomers plus Thapsigargin, mitochondria entered a fragmentation process, from where they did not recover (Figure 4Bc: white circle, 2 h: 0.25 ± 0.2 μm). These results indicate that RyR and SERCA are involved in calcium homeostasis and des-regulation of this homeostasis is protected by Wnt-5a through RyR clustering associated with the ER (Silva-Alvarez et al., submitted). Wnt-5a, a non-canonical Wnt/Ca^+2^ pathway, activates a physiological signal which triggers mitochondrial dynamics, protecting neurons from the neurotoxic effects of the Aβ oligomers.

### Wnt-5a prevents Bcl-2 increase induced by Aβo in mitochondrial compartments

**Figure 5:**
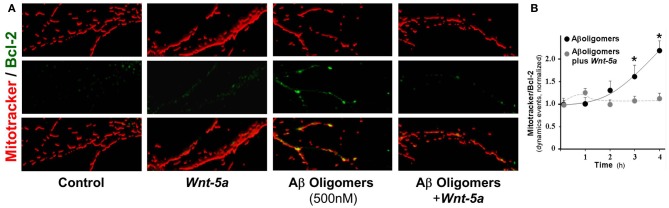
**Wnt-5a prevents Bcl-2 increase triggered by Aβo in mitochondrial compartments.** The micrographs show representative mitochondria stained with Mitotracker orange and Bcl-2 (green). **(A)** control neurons, neurons treated with Wnt-5a for 2 h, neurons treated with Aβ oligomers (500 nM) and neurons treated with Aβ oligomers plus *Wnt-5a*. **(B)** graph shows the co-localization of Bcl-2 staining over mitochondria (dynamic events) in the presence of Aβ oligomers with and without Wnt-5a. Primary cultures of rat embryo hippocampal neurons (15 DIV) were treated at 37°C. Results are the mean ± SEM. *n* = 4–5 experiments, the number of dynamics events were measured in 100 μm of dendrites. Student's *t*-test, ^*^p < 0.05.

To determine the role of Wnt-5a as a neuroprotective agent against Aβ o damage, we stained the mitochondria with Mitotracker and examined Bcl-2 levels, a Wnt-target gene and anti-apoptotic protein (Fuentealba et al., 2004; Fuenzalida et al., 2007), by immunofluorescence in neurons exposed to 500 nM Aβ o in the presence of Wnt-5a (Figure 5A). As expected, there was a weak positive reaction to Bcl-2 in control neurons (first row) and neurons treated with Wnt-5a (second row). Neurons treated with the Aβ oligomers showed an increase in Bcl-2 staining in the mitochondrial compartment (third row). This effect was almost completely prevented when neurons were co-incubated with Wnt-*5a* in the presence of Aβ o (fourth row). Similar experiments were carried out in a time-dependent manner in the presence of Aβ o, and an increase in the deposition of Bcl-2 on mitochondria was significant (Figure 5B: black circle, 3 h: 1.6 ± 0.3). It was apparent that soon after the application of Wnt-5a, Bcl-2 levels were normalized up to 2 h of treatment (Figure 5B: gray circle, 2 h: 1 ± 0.1). These results indicate that Wnt-5a modulates the apoptotic changes induced by Aβ o to prevent neuronal death.

## Discussion

Mitochondria are dynamic organelles that constantly fuse and divide. The correct modulation of the mitochondrial fission–fusion equilibrium is critical to regulating cell death, mitophagy, and organelle distribution (Chan, 2012). Excessive mitochondrial fission is associated with pathologies such as Charcot-Mary-Tooth-IIa, neurodegenerative diseases and diabetes (Detmer and Chan, 2007; Yoon et al., 2011; Itoh et al., 2013). In AD, it is possible to recognize several defects in mitochondrial functions (Wang et al., 2009), for instance, impaired mitochondrial fission or fusion, can produce oxidative damage (Kageyama et al., 2012) and may also produce local bioenergetics failure in neuronal processes lacking mitochondria. Currently, there is considerable evidence suggesting a key role for the morphological abnormalities and/or dysfunctional mitochondria in the pathogenesis of AD (Lin and Beal, 2006; Cho et al., 2010; Manji et al., 2012; Itoh et al., 2013), indicating mitochondria as a central organelle and a new potential therapeutic target against AD.

Mitochondrial dysfunction is a prominent feature on AD neurons. Quantitative morphometric analysis of mitochondria shows increased abnormal and damaged mitochondria in the AD brain (Hirai et al., 2001; Baloyannis, 2006). Aβ causes rapid and severe impairment of mitochondrial transport (Rui et al., 2006), and Aβ overproduction causes abnormal mitochondrial dynamics in neurons (Wang et al., 2008). Interestingly, Aβ oligomers caused mitochondrial fragmentation and reduced mitochondrial density in neuronal processes. Also Aβ oligomer-induced synaptic change correlates with abnormal mitochondrial distribution (Wang et al., 2009).

In a previous study, we demonstrated that activation of the canonical Wnt signaling pathway in hippocampal neurons prevents the neurotoxicity triggered by Aβ aggregates formed by amyloid fibrils (Alvarez et al., 2004). In the present study we showed for the first time that the activation of canonical and non-canonical Wnt signaling exerts a neuroprotective effect against Aβ oligomers at different levels: (1) firstly, the canonical Wnt/β-catenin pathway primarily affects the whole neuronal repertoire; (2) secondly, the non-canonical Wnt pathway through the Wnt-5a ligand specifically affects the mitochondrial compartment. In order to activate the canonical Wnt pathway we incubated hippocampal neurons with a GSK-3β inhibitor (6-BIO) (Meijer et al., 2004). In these studies, the pre-incubation with 6-BIO, in a concentration selected to inhibit GSK-3β (Polychronopoulos et al., 2004) prevented the decrease of β-catenin accumulation observed in neurons treated with Aβ oligomers, indicating that 6-BIO acts through the activation of the canonical Wnt/β-catenin pathway protecting neurons from Aβ oligomer toxicity. On the other hand, our previous work (Silva-Alvarez et al., submitted) together with these results, suggest that the non-canonical Wnt pathway activation, using Wnt-5a ligand, protects neurons against the toxicity induced by Aβ oligomers, producing a regulation of calcium homeostasis, which is altered by the presence of Aβ oligomers.

Both Wnt/Ca^2+^ and Wnt/JNK, activated by the Wnt-5a ligand, modulate NMDA receptors (Cerpa et al., 2010, 2011, Varela-Nallar et al., 2010). Furthermore, Wnt-5a signaling via the Wnt/Ca^2+^ pathway stimulates dendritic spine morphogenesis in hippocampal neurons (Varela-Nallar et al., 2010). Wnt-7a, a canonical Wnt ligand, also has an effect in dendritic spines (Ciani et al., 2011). Finally, *Wnt-5a* regulates inhibitory synapses, inducing the surface expression and maintenance of the GABAA receptor in the membrane of hippocampal neurons as well as the recycling of functional GABAA receptors through activation of CaMKII (Cuitiño et al., 2010).

**Figure 6:**
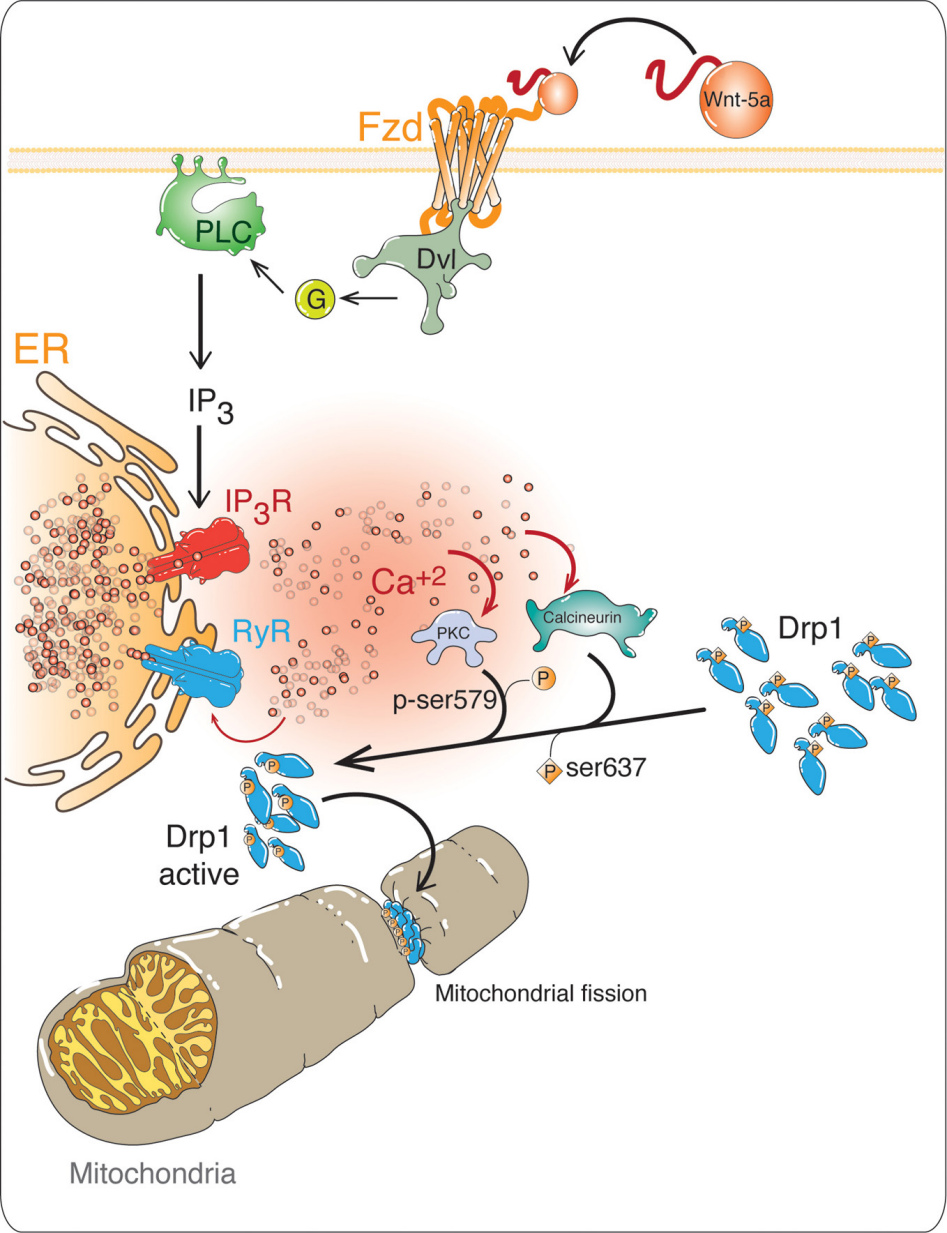
**Possible role of non-canonical *Wnt/Ca^2+^* pathway in mitochondrial dynamics.** In the non-canonical Wnt/Ca^2+^pathway, the binding of the ligand to its receptor Fz, activates Dishevelled (Dsh), which allows the activation of a trimeric G protein. The G protein activates phospholipase C (PLC), increasing the levels of inositol triphosphate (IP_3_), which increases the intracellular Ca^2+^ concentrations, coming from the ER. This Ca^2+^ induces further Ca^2+^ releases through RyRs. The high levels of Ca^2+^ activate Ca^2+^-dependent proteins such as protein kinase C (PKC) and the phosphatase Calcineurin. These enzymes regulate the activation of Dynamin-related protein 1 (Drp1), phosphorylation by PKC and CaMK and dephosphorylation by calcineurin, as a result Drp1 is translocated from the cytoplasm to the outer mitochondrial membrane (OMM), which is a signal for mitochondrial fission.

We recently established that Wnt-5a -induces mitochondrial dynamics in an ER-calcium related mechanism (Silva-Alvarez et al., submitted), suggesting that one of the underlying molecular mechanisms related to Wnt-5a effects might be due to the modulation of the balance between mitochondrial fission and fusion. The role of the Wnt/Ca^2+^ pathway in several neuronal processes has been well established (Kohn and Moon, 2005; Angers and Moon, 2009). In the Wnt/Ca^2+^ pathway, Wnt-5a can activate both PKC and CaMKII, by increasing the intracellular Ca^2+^ concentration coming from internal stores (Kuhl et al., 2000; Kohn and Moon, 2005). Considering that the ER is the main cellular calcium reserve (Bravo et al., 2012), the specific inhibition of RyR and SERCA studied here, suggests that Ca^2+^ certainly comes from the ER and supports the idea that ER-related calcium lies downstream of Wnt-5a effects, as illustrated in Figure 6.

The recovery of mitochondrial length observed in the presence of ryanodine, suggests that calcium from the ER, via RyR, contributes to the imbalance produced by Aβ oligomers leading to mitochondrial fragmentation as seen with the inhibition of RyR in the present study. The relationship between mitochondrial fragmentation and ER-mediated calcium release, has been previously reported in hippocampal neurons challenged with Aβ o (Paula-Lima et al., 2011), and with the direct agonist of RyR 4-CMC, that reproduced the mitochondrial fragmentation effects observed with Aβo (San Martín et al., 2012). There is strong evidence for the critical role of calcium balance on mitochondrial functions and fate. In this context, we have described that tetrahydrohyperforin (THH), a drug that prevents several toxic effects observed in a transgenic AD mouse model (Inestrosa et al., 2011), is able to avoid mitochondrial calcium overloading and at the same time can control alterations of mitochondrial fission-fusion dynamics in injured hippocampal neurons (Zolezzi et al., 2013). On the other hand, the anti-apoptotic Bcl-2 protein is a target gene of the canonical Wnt/β-catenin signaling pathway (Fuentealba et al., 2004; Fuenzalida et al., 2007; Youle and Strasser, 2008), which is activated in response to cell injury, such as exposure to Aβ oligomers or oxidative stress to prevent apoptosis. Several studies have revealed that Bcl-2 proteins could act on mitochondrial Ca^2+^ homeostasis on neural cells, allowing for more mitochondrial Ca^2+^ uptake without any mitochondrial respiratory impairment, suggesting that Bcl-2 can protect mitochondria from Ca^2+^ overload, although no consensus exists concerning the mechanisms underlying this function (Murphy et al., 1996; Zhu et al., 1999). In this direction, our results show that Wnt-5a is able to modulate the increase in the anti-apoptotic protein on the outer mitochondrial membrane of neurons, suggesting that Wnt-5a protects neurons from the early apoptotic effect induced by Aβ oligomers.

We conclude that the neuroprotective properties of Wnt-5a not only affect mitochondrial dynamics, but also might control molecular mechanisms related to apoptotic processes that take place in the living mitochondria.

Considering the importance of the regulation of intracellular calcium levels mediated by the activation of the Wnt/Ca^2+^ pathway, we propose a model for the possible mechanism of action of *Wnt-5a* on mitochondrial dynamics (Figure 6). In this model, Wnt-5a binds to the Fz receptor activating phospholipase C, which generates inositol-3-phosphate (IP_3_) and binds to its receptor (IP3 R) releasing intracellular Ca^2+^ from the ER. This Ca^2+^ could activate the RyRs and induce the Ca^2+^ release (Fill and Copello, 2002). In a previous work, we established that mitochondrial dynamics induced by Wnt-5a, was abolished by the use of the ER-calcium specific inhibitors of IP3R and RyRs, Xestospongin C and high concentrations of Ry respectively, supporting the idea that ER-related calcium is part of the signal transduction of Wnt-5a required to induce mitochondrial dynamics (Silva-Alvarez et al., submitted). The increased calcium activates several calcium dependent proteins such as PKC and Calcineurin. Our model suggests that these enzymes could activate Drp1, which controls mitochondrial fission by PKC-phosphorylation at Ser579 (Qi et al., 2011) and Calcineurin-dephosphorylation at Ser637 residues (Cereghetti et al., 2008), triggering Drp1 translocation from the cytoplasm to the mitochondrial outer membrane inducing mitochondrial fission.

Our research indicates that non-canonical Wnt signaling, activated by Wnt-5a ligand, regulates mitochondrial dynamics and/or preserves the MMP and prevents the mitochondrial fragmentation induced by Aβ oligomers and the exposure of Bcl-2, an anti-apoptotic mitochondrial protein, suggesting that Wnt components might be used to control mitochondrial dysfunction, such as the one observed in AD.

Considering that neurons are among the most energy-consuming cell types, it is essential to keep a stable metabolic-energetic stage during synaptic function, through normal mitochondrial functions. Therefore it becomes important to further study the role of the Wnt-5a ligand in mitochondrial functions in order to consider the mitochondria as a possible therapeutic target for AD treatment.

### Conflict of interest statement

The authors declare that the research was conducted in the absence of any commercial or financial relationships that could be construed as a potential conflict of interest.
